# Risk of cardiac implantable device malfunction in cancer patients receiving proton therapy: an overview

**DOI:** 10.3389/fonc.2023.1181450

**Published:** 2023-07-04

**Authors:** Milad Mirzaei, Pejman Rowshanfarzad, Suki Gill, Martin A. Ebert, Joshua Dass

**Affiliations:** ^1^ Department of Radiation Oncology, Sir Charles Gairdner Hospital, Nedlands, WA, Australia; ^2^ Department of Medical Imaging and Radiation Sciences, School of Biomedical Sciences, Monash University, Clayton, VIC, Australia; ^3^ School of Physics, Mathematics and Computing, The University of Western Australia, Crawley, WA, Australia

**Keywords:** proton therapy, cardiac implantable electronic device, malfunction, secondary neutrons, pacemakers, implantable cardioverter defibrillators, implantable loop recorders, pacing leads

## Abstract

Age is a risk factor for both cardiovascular disease and cancer, and as such radiation oncologists frequently see a number of patients with cardiac implantable electronic devices (CIEDs) receiving proton therapy (PT). CIED malfunctions induced by PT are nonnegligible and can occur in both passive scattering and pencil beam scanning modes. In the absence of an evidence-based protocol, the authors emphasise that this patient cohort should be managed differently to electron- and photon- external beam radiation therapy (EBRT) patients due to distinct properties of proton beams. Given the lack of a PT-specific guideline for managing this cohort and limited studies on this important topic; the process was initiated by evaluating all PT-related CIED malfunctions to provide a baseline for future reporting and research. In this review, different modes of PT and their interactions with a variety of CIEDs and pacing leads are discussed. Effects of PT on CIEDs were classified into a variety of hardware and software malfunctions. Apart from secondary neutrons, cumulative radiation dose, dose rate, CIED model/manufacturer, distance from CIED to proton field, and materials used in CIEDs/pacing leads were all evaluated to determine the probability of malfunctions. The importance of proton beam arrangements is highlighted in this study. Manufacturers should specify recommended dose limits for patients undergoing PT. The establishment of an international multidisciplinary team dedicated to CIED-bearing patients receiving PT may be beneficial.

## 1 Introduction

Proton Therapy (PT) is a highly advanced and promising form of particle therapy (1). PT has the potential for substantial healthy tissue sparing and can potentially increase survival and reduce toxicity in selected patients including those with malignant tumours of the thoracic region. Two comparative studies have investigated the efficacy of PT in oesophageal cancer patients and described noticeable enhancements in both progression-free survival and overall survival across 1 to 5 years compared to photon- external beam radiation therapy (EBRT) (2, 3). Xi et al. (2) demonstrated that 150-250 mega-electron volt (MeV) PT offered a superior target dose coverage, and more importantly, delivered a significantly lower dose to the heart compared to 6-18 megavolt (MV) photon-EBRT, representing 11.6 Gray Equivalent (GyE) versus 19.9 Gy respectively. Moreover, the 5-year follow-up revealed that patients who received PT had a significantly higher progression-free (34.9% versus 20.4%) and overall survival (41.6% versus 31.6%) compared to those irradiated with photon-EBRT.

The progressive increase in the aging population and the existence of common risk factors between cardiovascular disease and cancer means that a larger number of patients with cardiac implantable electronic devices (CIEDs) will require EBRT (4–7). However, studies have found that ionising radiation delivered during EBRT can cause malfunctions in CIEDs (8–12). These malfunctions appear to be related to device cumulative radiation dose, and interactions of secondary neutrons with the device particularly at photon energies ≥10 MV and electron energies of ≥ 20MeV. However, CIED malfunctions as a result of electromagnetic interference (EMI) have also been previously reported during photon-EBRT (13). The influence of EMI generated by linear accelerators vary in different modalities (14–16). PT not only produces a greater number of secondary neutrons, but also has a more complex EMI compared to linac-produced electron or photon beams, and may trigger sudden CIED malfunctions (17, 18). EMI can also alter the voltage in the cardiac pacing leads (19).

The effects of neutron radiation on the electrical properties of semiconductors, including damage and degradation in performance of diodes, integrated circuits, MOSFETs and batteries has been reported. It can also introduce pseudo-electric signals and pulses (20–23).

**Figure 1:**
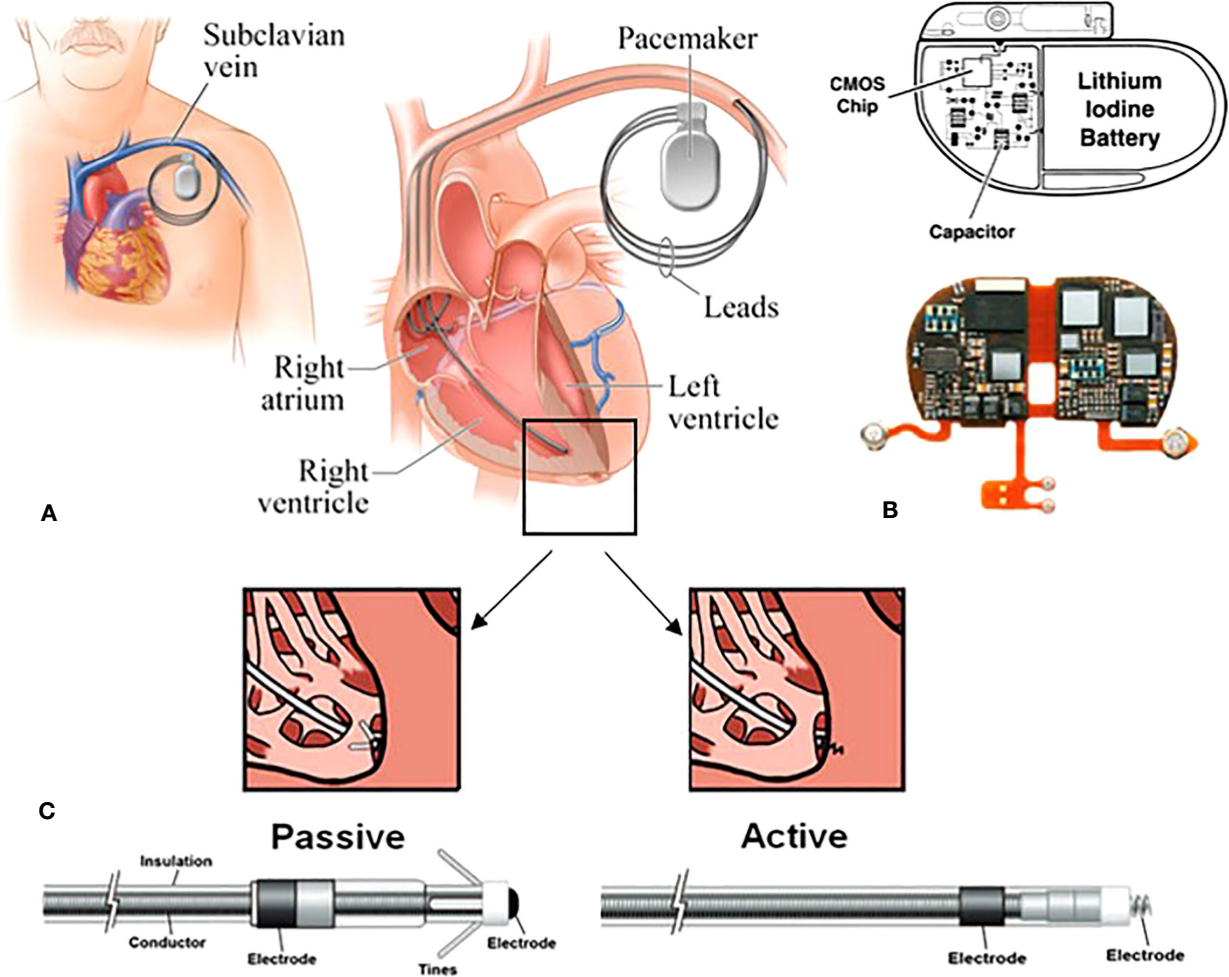
Schematic of a transvenous PM system consisting of the main device and the pacing leads. **(A)** in this approach, the leads are implanted endovascularly within the right atrium, and the right/left ventricles. **(B)** the main device comprised of a hermitically sealed titanium or stainless-steel case encompassing the battery and the circuitry. The reed switch, capacitors and resistors are intricately linked to complementary metal-oxide semiconductor (CMOS) chips. **(C)** two types of fixation mechanisms: the electrodes are attached to the appropriate chambers via tines (passive fixation) or screw (active fixation) (25–27).

Given the potential for the use of PT for most diseases, some consideration needs to be given to the presence of CIEDs. The most common CIEDs, cardiac pacemakers (PMs; leadless or composed of 1-3 pacing leads), are inserted in the subcutaneous tissue of the upper chest region, normally the infraclavicular area, where they monitor and help control cardiac arrythmia. The pacing lead(s) are generally implanted either percutaneously or *via* venous cutdown inside the atria, ventricles or coronary sinus of the heart (24), see **Figure 1**. PMs are specifically programmed to constantly monitor and deliver electrical impulses on demand to the heart chambers in patients with bradycardia arrythmias (28). The frequency of pacing entirely depends on a patient’s underlying cardiac rhythm, dividing patients into pacing dependant and non-dependent categories (29). Implantable cardioverter defibrillators (ICDs) incorporate the functionally of PM with the ability to deliver simulated electrical shocks to the cardiac muscle which allows detection and correction of patient’s ventricular tachycardia arrhythmias (28, 29). ICDs are implanted in patients at higher risk of sudden cardiac arrest and they reduce the risk of mortality by providing anti-tachycardia pacing and defibrillation (28). Having said that, cardiac resynchronisation therapy (CRT) devices have also been developed to further support these high risk patients. In addition to the above, implantable loop recorders (ILRs) (also known as insertable cardiac monitors) are USB-sized diagnostic devices that are implanted subcutaneously in the left pectoral area (30). They continually record electrocardiogram (ECG) to examine syncope, palpitations, and other inexplicable cardiac symptoms for up to three years (29, 30).

In patients who are pacing-dependant, damage to the CIED can cause shortness of breath, vertigo, syncope and may lead to cardiac arrest (31–34). The American Association of Physicist in Medicine Task Group 203 (AAPM TG-203) report highlights that even temporary CIED malfunctions resulting from dose rate effects can cause vertigo and syncope in pacing-dependent patients (33). This report recommends that patients with a CIED dose of >5 Gy or those receiving neutron producing beams should be monitored in the high risk category. A report for management of photon-EBRT patients with CIEDs was published by the Japanese Radiation Research Society and Japanese Society for Radiation Oncology (JASTRO/JCS) in 2021 (17). It classified patients receiving photon energies of ≥10 MV or CIED dose of >10 Gy as high risk regardless of their pacing dependency status. Discussion with a cardiologist before commencing treatment is recommended especially regarding the frequency of CIED function checks, intra-fractional monitoring, and even consideration of temporary external cardiac pacing during irradiation if required.

To further mitigate CIED malfunctions, manufacturers have been utilising more safety measures in their products; for example, hermetically-sealed metal shielding, signal filtering and nanomagnetic insulation (25, 35). Despite these improvements, several studies (36–44) have indicated that CIED malfunctions can occur via direct or indirect proton beams and their secondary neutron particles manifesting a series of hardware and software malfunctions. Hardware malfunctions represent permanent irreversible damage (including battery issues) to the hardware of the device and require replacement (17, 45). Software malfunctions are classified into: a) power-on reset (POR) which in most cases requires reprogramming by a technician (the most critical software error, overwrites the programmed data and reverts back to the safety backup mode/default factory setting) b) partial electrical reset (PER) which does not require reprogramming (the moderate software error, such as incomplete loss of memory) and preserves the programmed pacing mode c) transient or minor error (TE) which occurs only during irradiation (such as under- and over-sensing, noise) which is recorded in the device data log and only detectable during interrogation (37, 45, 46).

Previous reports have stated that inclusion of the main body of a CIED in the direct proton beam should be avoided (17, 33). The primary cause of CIED malfunctions during PT appears to be the increased production of secondary neutrons and their interactions with various components of CIEDs, particularly the complementary metal-oxide semiconductor (CMOS) chip. During PT, secondary neutron particles are produced due to the inelastic collision of incident protons with the atomic nuclei of the patient or beam modifying devices (*e.g.*, collimators, range modulators, and compensators) (47). The amount of neutron production predominantly depends on PT delivery modes, radiation energy and different beam lines/manufacturers (48). For instance, in the passive scattering (PS) mode, a larger number of neutrons are inevitably scattered both inside the treatment room and within the patient body (due to a greater number of beam modifiers) increasing the risk of secondary malignancies (48, 49). In the pencil beam scanning (PBS) mode, neutron production is substantially reduced outside the patient’s body, yet a considerable number of neutrons are still generated within the patient body (13, 50).

CIED malfunctions have been previously detected during high energy photon-EBRT as the result of following:

1) Nuclear reaction ^10^B(n,α)^7^Li, resulting from the interaction of neutrons with boron-10 used in BPSG (borophosphosilicate glass) of dielectric layers used on CMOS which generates α particles that disrupt electric current (51, 52);
2) Loading the silicon and the silicon dioxide (SiO_2_) insulator of CMOS with excess electron-hole pairs further accumulating a net positive charge on the insulator which can result in aberrant electrical pathways (14, 53, 54);
3) Ionisation of internal CIED contents which generates static field between the main body of CIED and its inner circuitry; which then gradually dissipates by leakage currents inside the CMOS (15, 55);
4) Interaction of scattered neutrons with the hydrogen-rich elements covering the CMOS which disturbs the circuitry (56);
5) Radiation-induced structural damage to the CIED pacing leads (composed of high-Z materials) resulting in shock coil failure (57).

Practical guidelines and protocols have speculated the impact on CIEDs of delivering PT based on small cohort studies and mainly the neutron producing characteristics of electron- and photon beams (31–34, 58, 59). However, the secondary neutrons produced during PT have significantly higher energies (*i.e*., greater penetrating power) compared to those generated during the former types of EBRT (48). Hence, it is extremely important to consider such discrepancies when establishing the safety of PT for CIED-bearing patients. This study has been conducted to quantify the risks associated with CIED malfunction in cancer patients undergoing different modes of PT and to provide recommendations for future practice and reporting.

## 2 Methods

A search was performed in November 2022 using the PubMed and the Web of Science databases to obtain available work written in the English language reporting on CIED malfunctions in patients treated with PT. The search included the following article types: systematic reviews, meta-analyses, randomised controlled trials, case series, experimental and observational studies between January 2002 and November 2022. The search details were as follows: “proton therapy” OR “proton beam” AND (“cardiac device” OR “implantable” OR “defibrillator” OR “pacemaker” OR “loop recorder”) AND (“malfunction” OR “error” OR “failure”). The publications were screened to find pertinent studies based on their title and abstract, searching for keywords such as ‘proton therapy’, ‘cardiac implantable device’, ‘malfunction’, and ‘neutrons’. All *in vivo*/*in vitro* studies investigating the impact of PT on CIEDs were considered for inclusion. Studies utilising fast neutron therapy, proton- or neutron-boron capture therapy, hadron ion therapy (*e.g.*, helium, carbon) and other types of EBRT such as photons and electrons were excluded given their distinct physical properties. This study was performed in accordance with PRISMA (60). Data extraction was conducted by the corresponding author.

This review was designed to systematically classify and evaluate all recent studies investigating CIED malfunctions and their associated risks in cancer patients undergoing PT. In this review, the word ‘risk’ refers to the probability of cardiac symptoms which may arise during or post PT delivery as a result of CIED malfunctions. Notably, there were no randomised controlled trials that measured these risks in this patient population. Thus, the references of selected articles were manually screened for additional practical guidelines and reports based on clinical relevance.

## 3 Results

**Figure 2:**
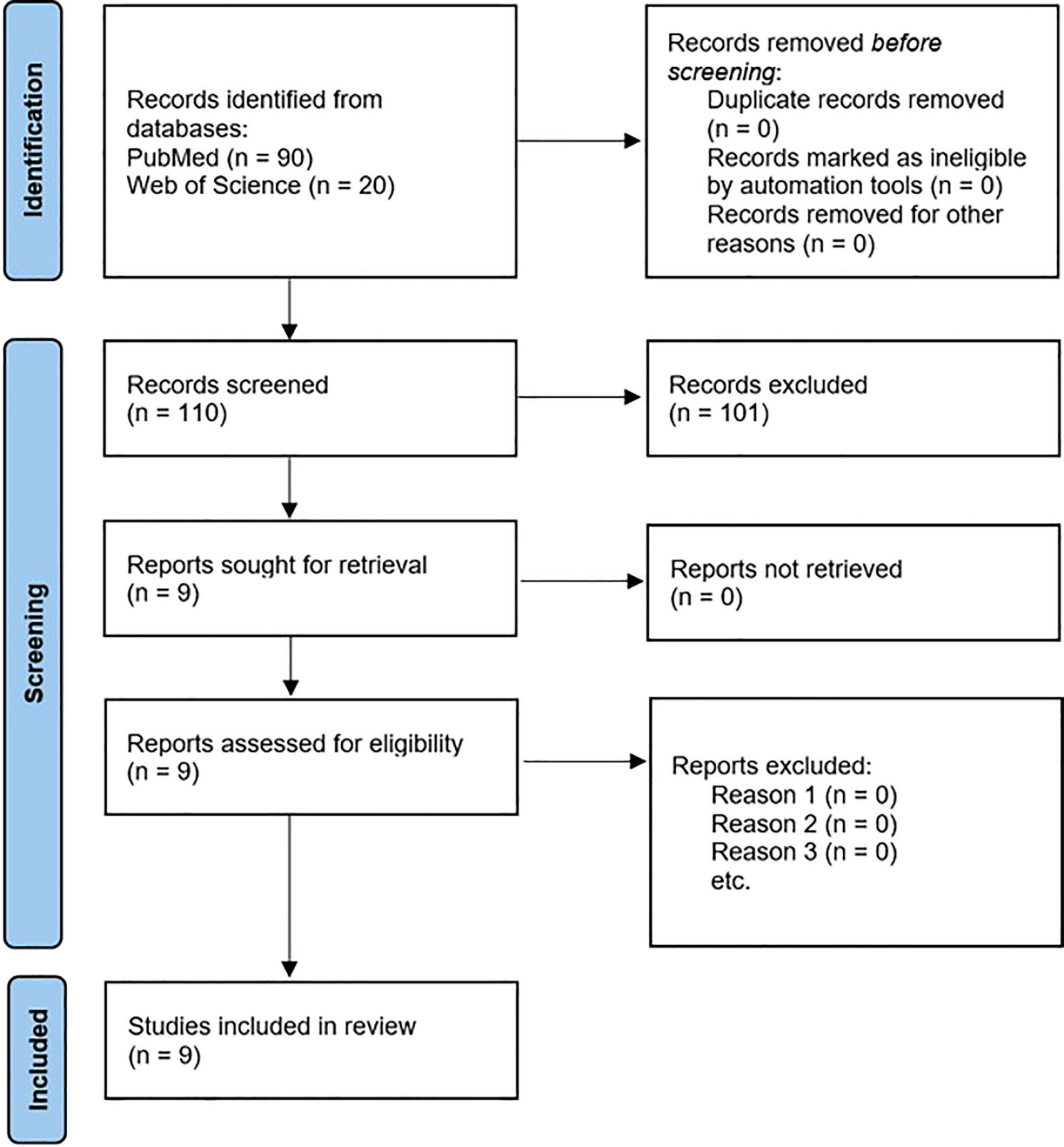
PRISMA flow chart of the selection process for articles to review (60).

The PubMed and the Web of Science databases retrieved a total of 110 publications that were subsequently assessed for eligibility. Assessment of these publications is depicted in **Figure 2** using the flowchart by PRISMA (60). One hundred and one articles were excluded as they did not suit the inclusion criteria for this review. Wootton et al. (61) was not included in the table of results because their study focused on proton dose perturbations caused by the presence of an in-field CIED pacing lead (*i.e.*, the effect of pacing lead on PT dose delivery) rather than assessing the lead’s functionality during or post PT. The references of the nine remaining articles were thoroughly screened for additional publications. The investigation identified a total of nine articles (36–43) which met the inclusion criteria for this review. Details of these studies are described in **Supplementary Table 1**.

### 3.1 *In vivo* studies

Five retrospective studies (36, 38–40, 42), from April 2001 to 2021, provided reports of a total of 114 cancer patients with CIED (age range between 66 and 90 years old) having received PT in ten institutions worldwide. Information about patient characteristics, cancer types and PT modes are listed in **Table 1**.

**Table 1:** Patient characteristics and cancers.

Characteristics	Patients
Treatment modality	
Proton therapy	114
Proton therapy mode	
Passive scattering	94
Pencil beam scanning	20
Gender	
Male	18
Female	3
Unspecified	93
Median age	76 (range 66 – 90)
Pacing dependency status	
Dependent	10
Non-dependent	40
Unspecified	64
Cancer type	
Head and neck cancer	
Submandibular carcinoma	1
Laryngeal carcinoma	1
Skull base carcinoma	1
Thoracic cancer (lung, oesophagus, thymus)	
Lung carcinoma	20
Other (unspecified)	27
Abdominal cancer	
Pancreatic carcinoma	1
Hepatocellular carcinoma	10
Liver (unspecified)	19
Pelvic cancer	
Prostate carcinoma	25
Unclassified cancer	9

94/114 patients (82.5%) were treated with PS mode, the remaining 20/114 (17.5%) received PBS mode. There were 18 males, 3 females, and 93 unspecified genders. Of 114 patients, 96 patients (84.2%) carried a PM, 17 patients (14.9%) had an ICD, 1 patient (0.9%) a CRT-D, and none had an ILR. Only one study (36) specified the implanted year of CIEDs. Information about the presence of CIED pacing leads were provided only by two studies (36, 39); 6/96 PMs definitely had pacing leads while the remaining devices could not be confirmed.

In terms of pacing dependency status, 10 patients (8.8%) were classified as pacing-dependent, 40 (35.1%) as non-dependent while the remaining 64 patients (56.1%) could not be identified. Overall, 47 patients (41.2%) received PT to the thorax (all except 2 patients were treated by PS mode), 30 patients (26.3%) to the abdomen, 25 patients (21.9%) to the pelvis, 3 patients (2.6%) to the head and neck region, and 9 patients (8%) were unclassified.

PT energies were not reported in two studies (38, 40). In other studies (36, 39, 42), the PT energy ranged from 150 to 250 MeV with the median energy at approx. 200 MeV. With the limited information available, the median prescribed treatment dose was about 66.0 GyE (range 36.6 – 88.0 GyE), while dose per fraction ranged from 2.0 to 6.6 GyE. None of the studies except for Oshiro et al. (36) reported the PT dose rate. In their study, the median dose rate was 2.45 GyE min^-1^ (range 2.06 – 3.00 GyE min^-1^).

Based on the available data, most of the CIEDs were manufactured by St. Jude Medical (7 PMs), Medtronic (6 PMs, 1 CRT-D), Biotronik (2 PMs, 1 ICD), Boston Scientific (2 PMs). In addition, there were two PMs (1 Guidant, 1 Intermedics) and other unknown CIEDs (3 PMs, 2 ICDs). Except for Hashimoto et al. (42) that did not clarify whether they maintained the manufacturer recommended CIED dose limits; all studies avoided direct irradiation of CIEDs, and the maximum doses to the device were restricted to ≤ 2.0 GyE.

Hardware malfunctions were not observed. A total of 21 software malfunctions (electrical resets and over sensing) were reported in 94 patients that were treated by PS-PT, and no malfunction occurred in the remaining 20 patients that received PSB-PT (overall incident 18.4% of patients treated with PT). Of 14 malfunctioned CIEDs, four PMs and one ICD had multiple electrical resets at different fractions. Six PMs, one ICD, and one CRT-D had a single reset each, and one ICD had three TE.

**Figure 3:**
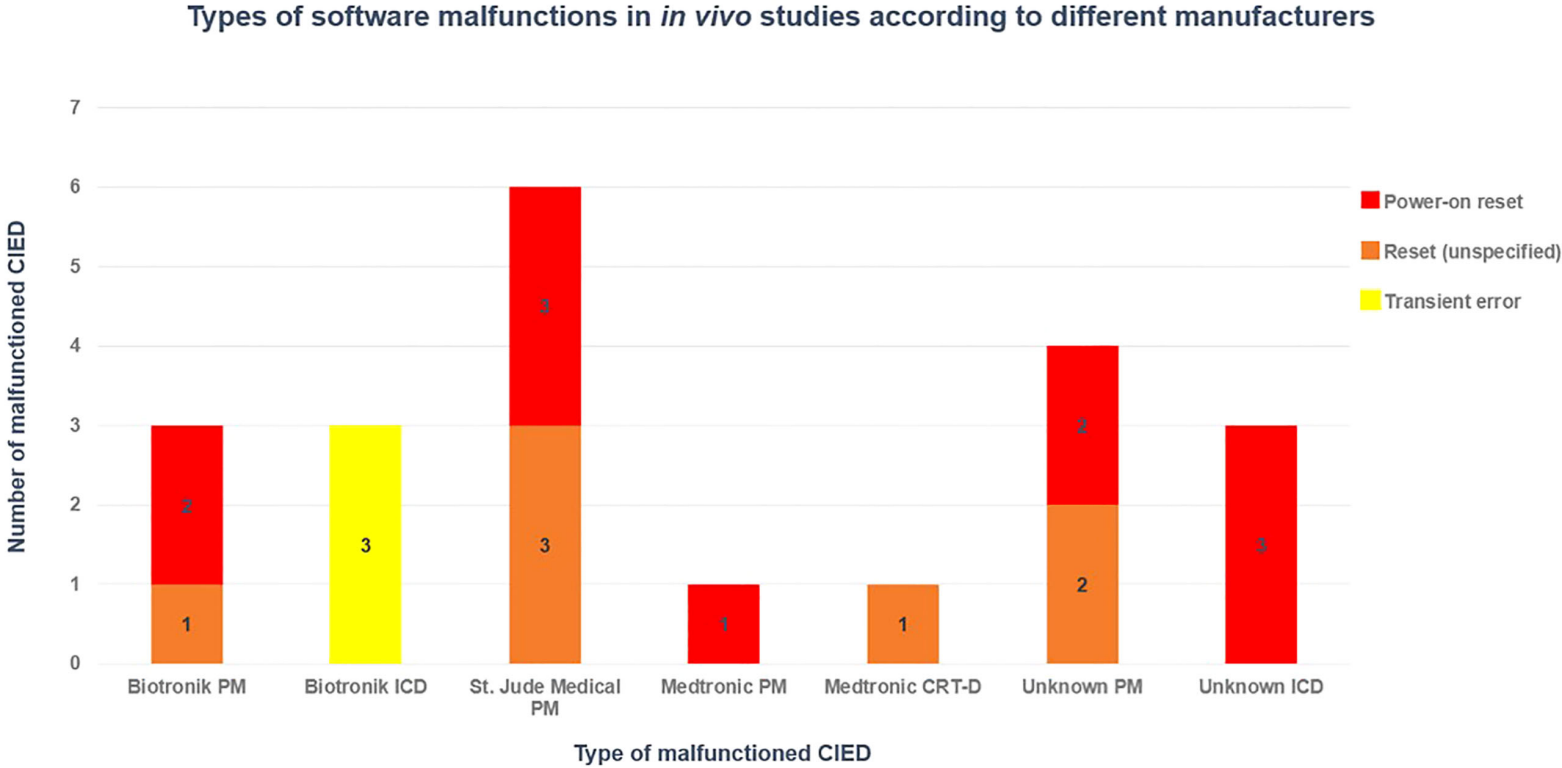
Distribution of software CIED manufacturers in different CIED manufacturers, based on *in vivo* studies (36, 38, 39, 42).

As shown in **Figure 3**, it has been concluded that the majority of software malfunctions (85.7%) were electrical resets (11 PORs, 7 unspecified resets) and the remaining 3/21 (18.3%) were of TE type. All CIEDs were successfully reprogrammed when necessary and they continued functioning properly post interrogation.

It is important to note that, Gomez et al. (38) reported an elective replacement indicator (ERI) event (*i.e.*, battery change message due to decreased battery voltage) in one ICD which was predicted in their pre-treatment interrogations, hence was not caused by PT. Seidensaal et al. (40) reported an increased impedance of the CIED pacing leads (fluctuation) in one patient during treatment, but they clearly stated that it already existed prior to treatment. Therefore, in order to avoid potential errors, these two pre-existing events were not included in the evaluations.

Overall, none of the retrospective studies (36, 38–40, 42) reported any morbidity or mortality. PT was safely delivered to all patients, and they remained asymptomatic in the event of software malfunctions. The majority of malfunctions were observed in thoracic patients 8/47 (18%) and upper abdominal 5/30 (16.7%), followed by head and neck 1/3 (33.3%). The overall incident of malfunction according to the CIED type was 10/96 (10.4%), 3/17 (17.6%), 1/1 (100%) for the PM, ICD and CRT-D respectively.

None of the five retrospective studies (36, 38–40, 42) specified PT beam arrangements. Most studies lack information essential to fully understanding the situation (*e.g.*, PT energy, dose rate, field size, presence of pacing leads within field, CIED to field distance, CIED model/manufacturer, CIED implanted year, proton/neutron doses to the CIEDs and leads).

### 3.2 *In vitro* studies

To date, three *in vitro* studies (36, 37, 41) have investigated the effects of PT on CIED malfunctions. A total of 66 CIEDs (26 PMs, 22 ICDs, 12 CRT-Ds, 6 CRT-Ps) and 1 pacing lead were exposed to different modes of PT. Overall, 5 hardware malfunctions (battery power depletion) and 90 software malfunctions (69 PORs, 7 PERs, 14 TEs) were identified in these devices. Remarkably, the hardware malfunctions only occurred during PBS-PT.

Only one study (36) specifically investigated the impact of PT on CIED pacing leads. The researchers directly irradiated the pacing lead of a Medtronic PM with a 200 MeV PS beam while the lead was located at the ‘centre’ of the 10.0cm long spread-out Bragg peak (SOBP). The pacing lead received a total dose of 35 GyE. Their experiment revealed that direct PS beam to the lead did not alter the pulse intervals or voltage at that dose range.

Hashimoto et al. (37). observed a total of 29 software malfunctions (14 TEs, 8 PORs, 7 PERs) occurred over 40 fractions (incidence 73% per fraction) in four Medtronic ICDs. The POR events were presented by sudden device failures, altered pacing rate, and switched over to safety back up mode; while PER and TE events did not impact the function of devices. POR occurred at a rate of ~1 per 50 GyE, while ~1 software error was detected in every 15 GyE. The predicted ICD neutron dose per 1 GyE proton was ~2.7mSv (1.3-8.9 mSv/GyE inside phantom). They highlighted that these events were unpredictable and did not increase with accumulated radiation dose.

In the largest *in vitro* study, Bjerre et al. (41) reported a total of 5 battery depletions (all Medtronic ICDs) and 61 POR (60 Biotronik, 1 Boston Scientific) in 1728 fractions delivered using PBS mode. The overall incident of POR for PMs and ICDs was (2.3%, 2.5%), (0.7%, 2.1%) and (0.2%, 1.4%) per fraction at lateral distances of 0.5cm, 5.0cm, and 10.0cm, respectively. More specifically, in Biotronik devices, the probability of POR per fraction was estimated to be 19.4%, 5.1%, and 3.2% in the 0.5, 5.0, and 10.0cm setups, respectively. The risk of POR was significantly higher in ICDs than PMs in the 5.0 and 10.0cm setups. Except for one Boston Scientific PM which had a critical POR and was permanently locked in the safety mode; all Biotronik devices were successfully reprogrammed by technicians.

Furthermore, 14 CIEDs showed major battery fluctuations of which 9 devices had a complete battery recovery at 30-day follow-up. However, 5 Medtronic ICDs represented with clinically significant battery power depletion which was unrecoverable. Remarkably, TEs such as noise, pacing inhibition, over-sensing *etc.* did not occur during the live monitoring experiment of 13 CIEDs (with leads connected) receiving a total of 362 fractions. The estimated maximum neutron doses to CIEDs were 6.94mSv, 3.71mSv, and 1.91mSv per fraction in devices at 0.5cm, 5.0cm, and 10.0cm distances, respectively. Secondary neutrons increased the relative risk of POR in CIEDs by 55% per mSv.

### 3.3 Decision algorithm

Based on the maximum secondary neutron dose of up to 7mSv reported by the previous study (41), Stick et al. (43) developed decision algorithms to further improve clinicians understanding of selecting CIED-bearing patients with breast and head and neck diseases undergoing PBS-PT. Remarkably, none of the patients that received PBS-PT had a real CIED; instead, devices were retrospectively delineated in the planning system for dosimetric comparison.

For eight breast patients with contralateral virtual or real CIEDs, the clinical target volume (CTV) volumes ranged from 281cm^3^ to 2977cm^3^ (median 1264.5cm^3^). The distance from the CIED to CTV ranged from 8.4cm to 13.0cm (median 8.4cm). In patients that received 2 GyE/Fx, the maximum neutron dose to CIED ranged from 1.3mSv to 8.0mSv (median 4.9mSv); for the 2.67 GyE/Fx group, it was between 1.7mSV to 10.6mSv (median 6.5mSv); and for the 5.2 GyE/Fx group dose ranged from 3.4mSv to 20.8mSv (median 12.6mSv). The two breast patients with an ipsilateral real CIED had CTV volumes of 412cm^3^ and 955cm^3^ with CTV to CIED distances of 2.0cm and 3.1cm, respectively. The maximum neutron doses for the 2 GyE/Fx were 5.6mSv and 7.2mSv, for the 2.67 GyE/Fx were 7.4mSv and 9.6mSv, and for the 5.2 GyE/Fx were 14.5mSv and 18.6mSv.

For breast patients with contralateral CIED, the maximum neutron dose to CIED+5mm was less than 7.0 mSv for patients with CTV < 1500cm^3^ receiving 2 GyE/Fx, and similarly for those with CTV < 1000cm^3^ receiving 2.67 GyE/Fx.

For the five head and neck patients with ipsilateral virtual CIEDs, the device to CTV distances ranged from 2.7cm to 5.5cm (median 5.0cm). Maximum neutron doses to CIEDs were between 2.4mSv to 4.9mSv (median 2.7mSv), and for the CIED+5mm was below 7mSv.

## 4 Discussion

To the authors’ knowledge, this is the first review focusing on the influence of proton beams on different types of CIED malfunctions. Potential risks of PT for patients implanted with the new generation CIEDs (*i.e.*, fabricated with distinct insulating materials and intricate circuitry) is still ambiguous. Direct beams to the body of CIEDs should be avoided in PT (17, 33). Nonetheless, indirect proton beams can still generate significant amounts of scattered neutrons that can interfere with the sensitive electronic circuitry (the main contributing factor for CIED malfunctions) leading to potential cardiac symptoms in patients. Furthermore, the nuclear interactions between direct PT beams and the modern CIED pacing leads or electrodes at higher energies (>50 GyE) might also influence clinical consequences.

In the last 10 years, a limited number of studies have investigated CIED malfunctions during PT (36–43). The focus of these studies has always been on the sensitivity of PMs and ICDs to PT. Yet, the impact of PT on CRT and ILR devices as well as its potential effects on CIED leads and electrodes have not been thoroughly investigated. There is an inconsistency in the way some of these studies reported their results. Some failed to provide essential information such as proton energies, neutron dose to CIED, dose rate, field size, field-to-CIED distance, beam arrangements, and most importantly CIED model/manufacturer. Therefore, it is still questionable to what extent these studies are reliable, and if not, which aspects should be measured in future research.

The PS mode of PT has been the main focus of literature primarily because it is a commonly used modality, though it also leads to a greater secondary neutron production compared to PBS mode. A recent study proved that CIED malfunctions can still occur during PBS-PT even at 10cm distance from the edge of radiation field (41). Considering the risk of malfunctions can be significantly reduced by utilising PBS instead of PS mode (62, 63), remarkably, the CIED malfunction predictive model proposed by Matsubara et al. (64) revealed that device error might still occur even in prostate patients (CIED-to-field distance of 50cm) undergoing PBS-PT depending on the type of CIED.

### 4.1 Effect of proton beam arrangements on the neutron fluence in different modes of delivery

Considering the limited available data, the analysis in this study showed that 75% of CIED malfunctions during PS mode seem to have occurred at neutron doses of above 14.0 mSv/Fx at CIED-to-field distances of between 0.3 to 8.0cm. This is twice the maximum CIED neutron dose (6.94 mSv) reported in a PBS study, while the devices were located at 0.5cm distance from the beam (41). In PBS mode, neutron doses of >14.0 mSv/Fx were only reported in breast patients (CIED-to-CTV distances < 10.0cm) planned to 5.2 GyE/Fx (43).

Several studies have measured secondary neutrons in different modes of PT (62, 65–68). Proton beam arrangements have been shown to have a direct effect on the secondary neutron fluence and CIED malfunctions. Wang et al. (65). highlighted in their work that the magnitude of scattered neutrons in PS mode varies at different positions relative to the proton beam axis (θ). This means that neutron doses to CIED can fluctuate depending on the device angular position relative to the beam axis. Previous studies (66, 69) stated that secondary neutron doses in PS mode are higher for increasing angles. A linear correlation appears to exist between the position of secondary neutrons relative to the beam axis and their spectra at that position. Shin et al. (70) reported neutron doses of between 0.088 to 1.590 mSv/GyE in the direction of the beam axis (θ=0). But under similar conditions, it was found to be the highest (3.88 mSv/GyE) off the beam axis (θ=135) at 25cm from the isocentre. Their findings support Carnicer et al. (69) study which also claimed that secondary neutrons produced during PS mode have lower energies at angles closer to the beam axis.

It is true that PBS mode reduces the production of secondary neutrons by approximately a factor of ten further minimising the radiation dose outside of the treatment field (62). Nevertheless, neutron production is still considerable within the patient body and this production cannot be easily eliminated by shielding. A comparative study assessed secondary neutron doses to the fetus in two different head and neck PBS plans (50). Three proton beams with energies 72.5-146.9MeV were used in each plan. The beams (gantry angles ranging 50-300°) that were perpendicular to the patient body axis (couch angle 0) generated the least number of neutrons (1-2 μSv/Fx). While one of the beams called the “*vertex field*” (gantry 290°, couch 90°) resulted in a significantly greater magnitude of neutrons (41.1 μSv) compared to all the other beams combined (7x μSv). They concluded that this is largely due to the beam arrangements as neutron values were significantly greater at closer angles relative to the beam axis. Unlike PS mode, high energy neutrons generated during PBS appear to scatter in a more forward direction depending on beam energy (68).

In conclusion, it can be assume that reducing the production of secondary neutrons and consequently minimising CIED malfunctions might be clinically feasible by utilising appropriate beam arrangements with respect to organs at risk and dosimetry protocols for different treatment sites. Paganetti et al. (71) have suggested that certain beam angles should be avoided in proton treatment planning due to range uncertainties and clinical implications. They have emphasised that utilisation of beam angle as an optimisation parameter in an LET-based optimisation module could enhance proton treatment planning. Development of a beam arrangement optimisation algorithm may be beneficial for planning patients with CIEDs undergoing proton therapy. This optimisation strategy would provide the safe beam angles (coplanar/non-coplanar) and beam energies depending on the angular position of CIED relative to the beam axis; and exposure of CIED to maximum neutron fluence produced.

### 4.2 Cumulative proton dose and dose rate effects

The AAPM-TG203 claims that cumulative proton doses are concerning only when the CIED is located within the proton beam, and that dose rates <0.01 GyE min^-1^ should be considered as low risk (33). The analysis in the present work revealed that PT-induced malfunctions occurred at a wide range of proton doses, did not increase with cumulative doses and were highly dependent on secondary neutrons as being the primary cause of malfunctions. Bjerre et al. (41) found that the risk of malfunction during PBS-PT is independent of accumulated radiation dose up to 72 GyE but dependent on secondary neutron dose per fraction. Hashimoto et al. (42) stated in their recent paper that the incident of CIED malfunctions were relatively lower in total doses of <60 GyE during PS-PT. Interestingly, the analysis in the present work showed that more than 80% of malfunctions during PS mode have occurred at radiation doses of between 15 to 55 GyE.

Previous studies on photon-EBRT expressed concerns about the influences of high dose rate and cumulative dose on CIED malfunctions (16, 72, 73). Matsubara et al. (73) have categorised high dose rate and large cumulative dose as secondary and tertiary risk factors, respectively, while scattered neutrons are considered the primary cause. Cumulative radiation dose can generate irreversible critical malfunctions in CIEDs such as battery depletion (classified as hardware malfunction) and altered telemetry capability due to the accumulation of aberrant charges in the battery and electronic circuitry (72). Moreover, high dose rates can charge the circuitry of CIEDs to a higher voltage, further introducing pseudo signals which can cause TEs during irradiation. In a clinical setting, three TEs were reported during delivery of 155MeV PS-PT to a lung patient (70 GyE/35Fx) with Biotronik ICD which was located at 15-20cm from the treatment field (42). Hashimoto et al. (37) observed 14 TEs in four Medtronic ICDs but they utilised a much larger fractional dose of at least 10 GyE.

Unfortunately, only two studies (36, 37) concerning the effects of PT on CIED malfunctions reported dose rates (ranging 2-3 GyE min^-1^) for the malfunctioned devices. Regarding the relationship between dose rate and CIED malfunctions during photon-EBRT, Mouton et al. (74) reported 0, 4, 14 and 68 out of 96 PMs, for dose rates ≤0.2, ≤1.0, ≤4.0 and ≤8.0 Gy/min, respectively. These outcomes highlight that dose rate can also play an important role on determining the risk of CIED malfunction, considering it is highly dependent on the radiation source, energy, and type of linear accelerator. Hence, understanding the relationship between PT dose rates and the probability of CIED malfunctions can be quite complex and require further investigation. Of particular interest are the potential technical challenges in FLASH PT which seems to be a promising modality for future cancer treatment (75, 76).

### 4.3 CIED distance and field size

**Figure 4:**
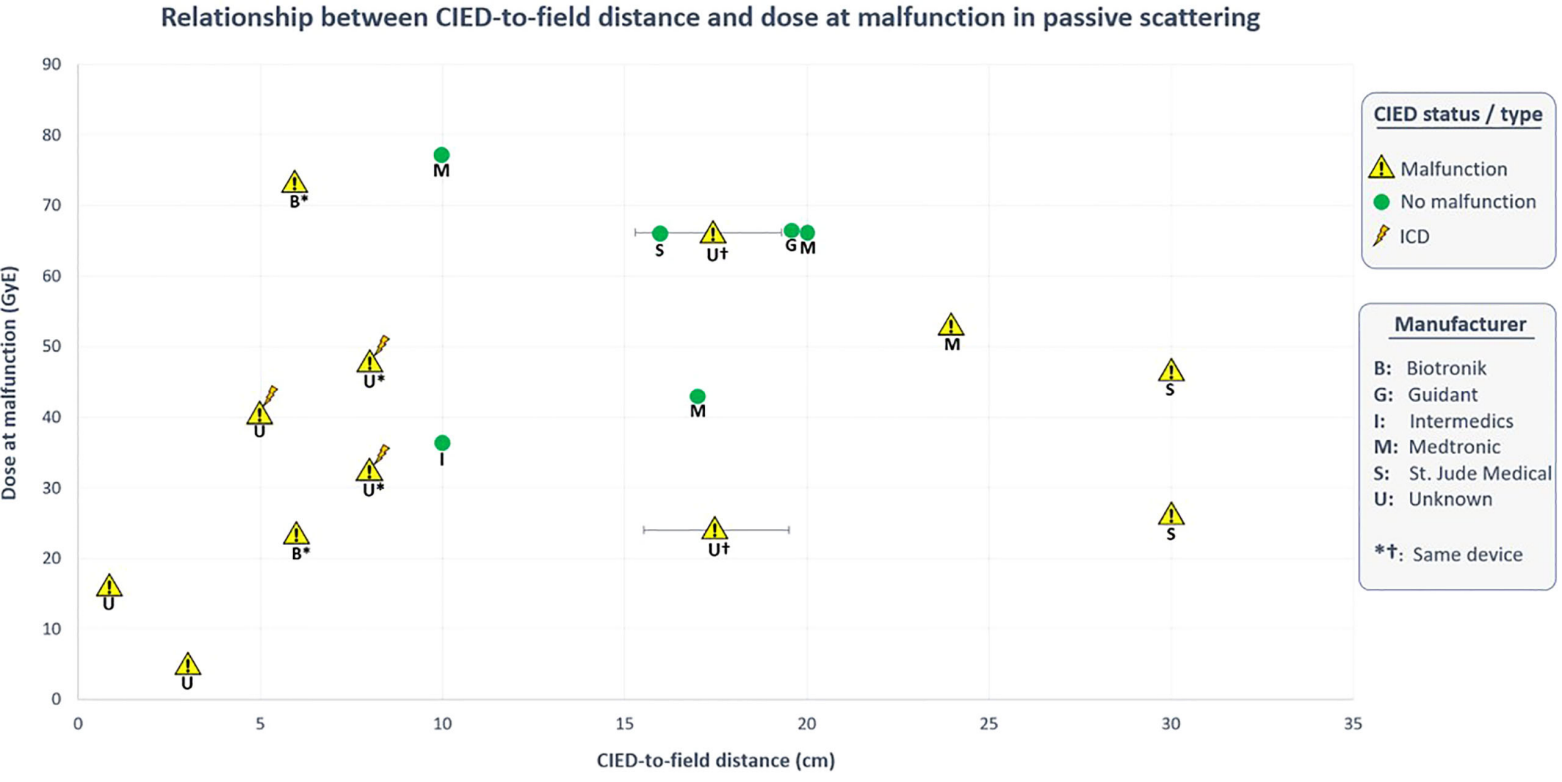
Relationship between CIED-to-field distance and dose at malfunction in PS-PT. Data obtained from *in vivo* studies (36, 38, 39, 42).

Hashimoto et al. (42) in their study stated that only 2 of the 15 lung patients (13.3%) treated with PS mode had CIED malfunctions; in both cases, the devices were located 15-20cm from the treatment fields. Only one of them (wearing an unknown PM) had electrical resets. Surprisingly, none of the seven patients with CIEDs located at shortest distances (0-15cm), or three patients with devices at greatest distances (20-25cm) experienced device malfunctions. Contrary to their results, none of the other *in vivo* studies concerning PS mode reported any malfunctioned devices at 10-20cm distance from the field. The present study revealed that 5/8 (62.5%) of these malfunctioned devices (3 PMs, 2 ICDs) were located at <10cm distance from the proton field, while the remaining 3 PMs (37.5%) at 24-30cm. It should be emphasised that apart from one unknown PM which discussed earlier, malfunctions were not observed at 10-20cm or >30cm distances (**Figure 4**).

Understanding the relationship between CIED-to-field distance and the probability of malfunction is quite complex in PS-PT. Undoubtably, the secondary neutron dose reduces with the distance from the edge of the field (77). With respect to the main contributing factors, it was revealed that malfunctions during PS mode have occurred at a median distance of 7 cm (range 0.3-30cm). This is the same median distance for devices with resets reported by Gomez et al. (38).

Furthermore, two studies (36, 39) have reported the distances of non-malfunctioned devices (all PMs, median 17.0cm). Remarkably, 8/10 of non-malfunctioned PMs (80%) were located at distances of 10-20cm, while the remaining two devices (20%) were at distances of >50cm. Hence, it is evident that CIED malfunctions in PS mode become quite significant at <10cm distance, particularly in ICDs. On the other hand, no malfunctions were observed in 20 patients treated by PBS mode (38, 40). Remarkably, more than half of these patients received treatment to the pelvis region meaning that CIEDs were located at greater distances (~50.0cm) from the treatment field. Guaranteeing the complete safety of PBS-PT for CIED-bearing patients is quite difficult considering such a small patient cohort. Interestingly, only one study (40) specifically reported the median distance from CIED to the planning target volume (PTV), and CIED to 10% isodose line which were 13.4cm (range 4.1–17.9 cm) and 11.6cm (range 2.4–17.1 cm), respectively. Nevertheless, in the largest *in vitro* study by Bjerre et al. (41), 60 out of 61 software malfunctions occurred in 13 devices from one manufacturer at distances of 0.5-10cm over 1728 fractions. All devices except one (Boston Scientific PM) were successfully recovered, they highlighted that treating patients with PBS is manageable and risks are acceptable considering the superior advantages of PBS mode.

In terms of field size in PS mode, Hashimoto et al. (40) found that larger field sizes are associated with increased device error (caused by secondary neutrons) but the test for trends was not statistically significant (*p* = 0.196). None of the other *in vivo* studies on interactions of PS mode with CIEDs have provided us with information about field sizes.

In PS-PT, the relationship between neutron yield and field size is influenced by factors such characteristics of the proton beam (and their energy) entering the treatment head, the modulator wheel, and the material in the double scattering system (77). It is evident that the treatment head is the major neutron source compared to the patient contribution (78). A significant percentage of proton beams may be attenuated in the patient-specific aperture/collimators; causing the number of secondary neutrons (and their energy) to be dependent on the ratio of proton field size and aperture (77, 78).

Mesoloras et al. (79) found that the neutron dose equivalent mSv/GyE decreases with increasing aperture size. Zacharatou-Jarlskog et al. (77) stated that the neutron yield from the treatment head typically decreases with increasing field size (*i.e*., as the field size gets larger, less blocking aperture material). However, in the patient, neutron yield increases with proton beam energy and treatment volume. Larger treatment volumes located deeper in the patient result in significantly higher neutron equivalent doses. Due to: a) greater proton beam energies are required to reach deeper target volumes, b) for treating a larger volume, greater proton fluence is required to cover the entire target with the specified dose.

According to Hashimoto et al. (42), the relationship between neutron yield and field size is complicated and it depends on the balance of contribution from the treatment head and from the patient. It is important to note that, even for the same facility, there are significant variations from field to field, due to the geometry of treatment head and how the beam characteristics in PS mode are patient field specific (79). On the other hand, in PBS mode, the use of a range shifter to cover superficial target volumes will result in an increased amount of scattered secondary particularly for larger clinical CTVs (43). Stick et al. (43) concluded that a thinner range shifter of <57 mm water equivalent thickness would reduce the number of secondary neutrons. However, they stated that the CTV volume and dose per fraction were the most significant contributors to neutron production in breast patients undergoing PBS-PT. Whereas, in head and neck patients with ipsilateral CIED, the distance between CIED to CTV play an important role in the amount of neutron production.

### 4.4 CIED manufacturers and proton-specific dose limits

As illustrated in **Table 2**, there is a discrepancy in the recommended CIED dose limits among manufacturers. Most importantly, these recommendations are based upon electron- and photon-specific energies and their distinct secondary neutrons rather than PT-induced neutrons. Previous studies have revealed that the risk of device malfunction is significantly higher in ICDs and CRTs compared to PMs due to their complex configuration and electrical circuitry. Interestingly, it was found in this study that ICDs and CRTs are more likely to have POR type malfunction. Moreover, the findings of this study are in agreement with previous reports in the literature in that the probability of malfunction varies depending on the type of manufacturer (41, 45).

**Table 2:** Electron- and photon-EBRT dose recommendations and specifications indicated by the most common CIED manufacturers (17, 45).

Manufacturer	Max PM dose	Max ICD dose	Max beam energy	Casing material	Lead Shielding of device	Battery chemistry (80–82)	Comments based on PT studies
Biotronik (83)	2Gy	2Gy	≤10MV No direct beam to device	Titanium	Yes	Li/MnO_2_ Li/SVO CFx	Biotronik devices particularly ICDs and CRTs have had the highest number of software malfunction during PT (41)
St. Jude Medical (84)	No safe dose	No safe dose	Not specified Do not use ionising radiation in the proximity of an implanted device	Titanium	Not specified	Li/SVO Li/SVO CFx	Some PMs had software malfunctions (36, 39, 42)
Medtronic (85)	5Gy	1–5Gy Depending on the model	≤10MV	Titanium	No (ineffective against neutrons	Li/SVO Li/SVO CFx	Software errors have occurred in these devices (37, 39, 42). 5 ICDs had significant loss of battery capacity (hardware malfunction) (41)
Boston Scientific (86)	No safe dose limit (Maximum total dose of 2Gy used as a reference)	No safe dose limit (Maximum total dose of 2Gy used as a reference)	Not specified	Titanium	Yes Consider all available shielding options	Li/MnO_2_ Li/SVO CFx	Only 1 PM reported which had a critical software malfunction (41)

PM, pacemaker; ICD, implantable cardioverter defibrillator; CRT, cardiac resynchronisation therapy device; PT, proton therapy; Li/MnO_2_, lithium-manganese oxide; Li/SVO, lithium-silver vanadium oxide; CFx, carbon monofluoride.

The present study shows that software malfunctions have occurred more frequently in Biotronik devices. Although Medtronic devices seldom had software malfunctions, their ICDs appear to be more susceptible to hardware damage such as battery depletion (41). Although, these findings are based on limited number of cases, however, this could be due to the battery chemistry which may contain certain elements (such as lithium) which interact with protons and neutrons (87).

This study investigates the possibility of proton-induced nuclear interactions in the hybrid batteries of Medtronic ICDs. These batteries contain Lithium-Silver Vanadium Oxide/Carbon Monofluoride or Li/SVO CFx in their structure (80–82).

Although the percentage composition of these elements may vary, the interactions of secondary neutrons with fluoride, oxygen, carbon, lithium, vanadium and silver could be significant which would adversely affect the patients undergoing PT. The expected interactions include,

[^18^F(n,α)^15^N] (88), [^16^O(n,α)^13^C] (89), [^12^C(n,α) ^9^Be] (90), [^7^Li(n,α)^4^Li] (91), [^50^V(n,p)^50^Ti] (92), [^109^Ag(n,p)^109^Pd] (93).

### 4.5 Impact of protons on pacing leads and electrodes

There is no completely safe PT dose for CIED pacing leads and electrodes. Since 2008, no studies have thoroughly examined the effects of PT on pacing leads and electrodes. Pacing leads with various geometries and made by different manufacturers have been considered as the “weakest link” of the CIED system by the specialists in the field (94). As the percentage of CIED-bearing patients requiring PT continues to increase, the robustness and functionality of these leads becomes extremely vital.

Pacing leads have been regarded as insensitive to radiation by previous reports (33, 44, 58). The pacing leads utilised in ICDs and CRTs are more intricate due to the defibrillation circuits (94). Shock coil damage of an ICD pacing lead has been previously reported for a breast cancer patient receiving 50 Gy photon-EBRT (57). A recent case report (95) demonstrated that high doses of photon-EBRT can be delivered safely to the bipolar lead of an ICD (Medtronic Sprint Quattro Secure; Model 6947); the maximum doses to the lead and the electrode were 55.72 Gy and 7.10 Gy respectively.

Unlike the old generation leads (*i.e*., unipolar) which exhibited under- and over-sensing issues, the modern “bipolar” leads seem to be less susceptible to non-cardiac signals (25, 27). The modern pacing leads are usually made up of a central core of silver, and an alloy (known as MP-35N) which is composed of cobalt, nickel, molybdenum, and chromium (25). While, the other parts of it such as the shock coils and the ring electrode are both made up of titanium, iridium, platinum and tantalum (61, 94).

In 2008, Oshiro et al. (36) in their *in vitro* experiment directly irradiated the pacing lead of a Medtronic PM (which was located at the centre of SOBP) up to a dose of 35 GyE using PS-PT. Although they showed that the pulse voltage/interval were stable during irradiation, but it is not clear which type of pacing lead (unipolar or bipolar) was used in their experiment. Later, in their *in vivo* study, only 4 of 8 patients with PMs (implanted >20 years ago) had pacing leads within the radiation field; of which only one of the PM leads (most likely Medtronic) in one patient was exposed to a maximum dose of 63 GyE. Furthermore, the lung cancer patient reported by Ueyama et al. (39) also had a Medtronic PM. Apparently, the lead was partially exposed to only one of the PS-PT beams, however they did not specify exactly how much of the prescribed dose (66 GyE) the lead received. With such limited studies particularly on ICD and CRT leads, it is not possible to foresee optimal functionality of pacing leads and electrodes from all manufacturers after being directly irradiated or exposed to higher doses, considering the commonly prescribed dose is 60-80 GyE in PT.

Remarkably, Wootton et al. (61) in their phantom experiment on proton dose perturbations showed that the nuclear interactions between the 200MeV PS beam and a Medtronic ICD lead’s high-voltage shock coils and the ring electrode (both composed of titanium, iridium, platinum, and tantalum) can vary significantly; depending on the movement and position of the lead relative to the SOBP (*i.e.*, proximal, central, distal locations). Consequently, the variable magnitude of proton energy loss in such high-Z materials (which differ among manufacturers) should be further investigated.

The analysis in the present study revealed that the nuclear interaction of protons and neutrons with some of the elements in CIED pacing leads such as chromium, tantalum, nickel, cobalt and titanium are not negligible. These interactions include,

[^52^Cr(n,p)^52^V] (96), [^181^Ta(p,5n)^177^W] (97, 98), [^58^Ni(n,p)^58^Co] (99), [^59^Co(n,p)^59^Fe] (99), [^48^Ti(n,p)^48^Sc] (99).

Considering that the pacing lead is in close proximity to the main body of CIED, it was assumed that interactions of the scattered particles produced during the above reactions with the electronic circuitry of CIED could potentially contribute to malfunction in the CIED.

In terms of PBS-PT, only one patient in the Seidensaal et al. (40) study had an increased impedance of the CIED leads, which fluctuated during irradiation. However, they clearly stated that it was present prior to commencing treatment. Bjerre et al. (41) in their real-time sub-study monitored 13 CIEDs (with leads connected) during irradiation and reported no abnormalities such as noise, under- or oversensing *etc.* However, it is important to note that all the pacing leads were outside of the direct PBS field. As Barcellini et al. (44) stated, previous PT studies on pacing leads lack important information about the number of leads particularly in CRT devices, which might potentially increase the probability of lead damages.

The complete functionality of pacing leads and electrodes at higher PT doses still seems ambiguous. Hence, the following steps should be considered in future reporting: a) specifying the details of pacing leads, b) contouring pacing leads and electrodes separately as an organ at risk in PT planning (100), c) reporting the mean and maximum doses of pacing leads and electrodes (95).

### 4.6 Miniaturised CIEDs

Advancement in nanoelectronics has empowered miniaturisation of CIEDs and their integrated circuits. Although, the new generation CIEDs have a lower power consumption, the major disadvantage of these tightly packed devices is the higher sensitivity to radiation damage (33). As an example, ILRs are USB-shaped, ECG recording devices that are used for long term monitoring of patients with arrythmias. Like other CIEDs, ILR has a battery, CMOS and subtle electrical circuitry that may also be affected by PT. Special electrodes are located at each end of these devices to enhance sensitivity to cardiac activity. Remarkably, it was noticed that no studies have examined the effects of ionising radiation on ILRs. Most manufacturers have not specified a safe radiation dose for ILRs. For instance, Medtronic has clearly stated that ionising radiation may trigger inappropriate episode detection or corrupt the data stored in the memory of Reveal LINQ ILR (101). ILRs have been given a radiation dose limit of 5 Gy before malfunction (18). But, they are expected to have a much lower dose tolerance particularly during PT due to their distinct fabrications and subtle circuitry.

## 5 Recommendations for the management of patients

The present review reveals that the incidence of CIED malfunctions in PT is variable. It is of great importance that the CIED-bearing patients undergoing PT are managed differently to those receiving other forms of EBRT. This is mainly due to the unique characteristics of protons and the secondary neutron fluence which appear to be variable depending on different modes of PT delivery and the treatment volume.

There is no doubt that direct proton beams to the body of CIEDs should be avoided. However, this review also recommends avoiding direct irradiation of the pacing leads and electrodes as far as practically possible for the reasons explained in Discussion sections 4.4 and 4.5. This emphasises the importance of a robust PT planning method in which appropriate beam arrangements are utilised for this patient cohort, see Discussion section 4.1.

It is highly recommended that all patients undergoing PT be closely monitored during treatment using an intra-fractional ECG or pulse oximeter, and comprehensive audio-visual system. Furthermore, a CIED functionality check is required for all patients before and after each fraction. In addition, a detailed analysis of the device data log should be performed after the first fraction and weekly after that. The need for a daily CIED check and remote CIED monitoring tools should be discussed with a cardiologist. Weekly and monthly CIED follow-ups are recommended for at least 6 months post completion of the PT course.

## 6 Conclusion

To the authors’ knowledge, this is the first review conducted specifically on PT-related CIED malfunctions. All CIED models (with or without pacing leads) exposed to PS and PBS modes of PT were compiled and evaluated. Remarkably, the effect of PT-induced EMI on CIEDs has not been investigated yet. However, it was found that a variety of CIED malfunctions particularly software errors are triggered by secondary neutrons and cumulative radiation doses in PT. Considering that very limited studies have been conducted on the exposure of new generation CIEDs and pacing leads to PT, the rate of malfunctions resulting from secondary neutrons and/or cumulative doses is unknown. PT beam arrangements and tissue heterogeneity are essential factors contributing to the secondary neutron fluence and CIED malfunctions. While CIED type and discrepancies of materials utilised in the manufacturing could be another reason for their sensitivity to radiation. Increase in CIED-bearing patients undergoing PT urges a call for further research in this field.

## Author contributions

Conception of study, MM and PR. Collecting literature, MM and PR. Writing manuscript, MM. Reviewing manuscript, MM, PR, SG, ME, and JD. All authors contributed to the article and approved the submitted version.
