# Supplementary material for: Risk of cardiac implantable device malfunction in cancer patients receiving proton therapy: an overview
Source: Front Oncol. 2023 Jul 4;13:1181450. doi: 10.3389/fonc.2023.1181450 (PMC10352826; doi:10.3389/fonc.2023.1181450)
Supplement: Supplementary file 1 [file Table_1.docx]

**Table 1.** *In vivo* and *in vitro* studies assessing the impacts of PT on CIEDs, published in English language since 2002.

| **Author / Year** | **Study type / size** | **CIED type / manufacturer / year implanted** | **CIED / tumour location** | **PT mode / energy (MeV)** | **Prescribed treatment dose (GyE/Fx)** | **Cumulative dose at malfunction (GyE)** | **Dose rate (GyE min^-1^)** | **Field size /**  **beam direction** | **CIED proton dose (GyE)** | **CIED neutron dose (mSv)** | **CIED to field distance (cm)** | **Type of CIED malfunction /**  **clinical consequences** |
| --- | --- | --- | --- | --- | --- | --- | --- | --- | --- | --- | --- | --- |
| Oshiro  et al.^36^  2008 | *In vitro* Observational*,* single institution  *In vivo*  *8 patients (8 PMs)* | 1 PM  **Kappa KSR 703; Medtronic, Minneapolis, MN* **(LIF)**  2 PMs  *Affinity DR; St. Jude Med / 2004*  *Phillos SR; Biotronik, Berlin, Germany / 1994* **(LIF)**  **Kappa KDR 721; Medtronic / 2003*  **Kappa KSR 700; Medtronic / 1984* **(LIF)**  **Kappa KDR 721; Medtronic / 2004* **(LIF)**  **Discovery II; Guidant / 1995*  **Virtus Plus II SR; Intermedics / 1995* **(LIF)**  **Solus-mini; St. Jude Med / 1989* | Placed between 2 phantoms  Liver  Lung  Liver  Liver  Liver  Liver  Liver  Liver | PS / 200  PS / 155–250  PS / 155–250  PS / 155–250  PS / 155–250  PS / 155–250  PS / 155–250  PS / 155–250  PS / 155–250 | 35GyE (to the section of lead in atmosphere), 15Gy (in saline)  66.0 / 10  72.6 / 22  66.0 / 10  42.9 / 19  77.0 / 35  66.0 / 10  36.3 / 11  66.0 / 10 | N/A  46.0 (Fx 7)  23.0 (Fx 7)  72.6 (Fx 22) | N/A  Median dose rate:  2.42  2.83  2.87  2.45  2.45  2.18  2.06  3.00 | 25x25x12.5cm^3^ */* 270° lateral beam  N/A  N/A  N/A  N/A  N/A  N/A  N/A  N/A | At 20GyE, a voltage reduction to approx. 1/2 after the lead  was placed in the saline  Max dose to lead:  N/A  6.6  N/A  13  63  N/A  0  N/A | N/A  12  6.0 | Lead placed at the centre of 10cm SOBP  30  6.0  N/A  17  10  20  10  16 | No change in PM pulse voltage or pulse interval  Hardware errors: Nil  Software errors: 3  POR  POR  POR  All PMs successfully reprogrammed / No reported morbidity or mortality |

CIED = cardiac implantable electronic device; ICD = implantable cardioverter defibrillator; LIF = lead in treatment field; PM = pacemaker; PBS = pencil beam scattering; PS = passive scattering; PTV = planning target volume; CTV = clinical target volume; PER = partial electrical reset; POR = power-on reset; SOBP = Spread-out Bragg Peak; TE = transient error; ERI = elective replacement indicator; * No malfunction

**Table 1.** Continued

| **Author / Year** | **Study type / size** | **CIED type / manufacturer / year implanted** | **CIED / tumour location** | **PT mode / energy (MeV)** | **Prescribed treatment dose (GyE/Fx)** | **Cumulative dose at malfunction (GyE)** | **Dose rate (GyE min^-1^)** | **Field size /**  **beam direction** | **CIED proton dose (GyE)** | **CIED neutron dose (mSv)** | **CIED to field distance (cm)** | **Type of CIED malfunction /**  **clinical consequences** |
| --- | --- | --- | --- | --- | --- | --- | --- | --- | --- | --- | --- | --- |
| Hashimoto  et al.^37^  2012 | *In vitro*  Observational, single institution | 4 ICDs  *Marquis DR 7274; Medtronic* | Placed on outside surface of a phantom  Patient bolus was not used | PS / 200 | 107.0 / 10 (range 2.0-20.0 Gy/Fx) | ~1 software error per 15.0 Gy  ~1 power-on reset per 50.0 Gy | 2.0 | 10x10cm^2^ regular field / 270° lateral beam | N/A | ~2.7 per 1Gy proton  Inside phantom: 1.3-8.9 per 1Gy proton | Distal end of the 6cm SOBP was 0.3cm inside and 3.0cm in front of CIED | Hardware errors: Nil  Software errors: 29 (8 POR, 7 PER, 14 TE)  All ICDs successfully reprogrammed |
| Gomez  et al.^38^  2013 | *In vivo*  retrospective, single institution  42 patients  (28 PMs, 14 ICDs) | 2 ICDs / 2 PMs  ICD  PM  PM  ICD | 23 thorax (oesophagus, lung, thymus);  15 prostate;  3 liver; 1 base of skull  Thorax  Thorax  Thorax  Thorax | PS (32 patients); PBS (10 patients)  PS / N/A  PS / N/A  PS / N/A  PS / N/A | Median 74.0  (46.8-87.5) / 35 (15-39)  74.0 / 37  50.4 / 28  60.0 / 30  87.5 / 35 | 40.0 (Fx 20)  16.2 (Fx 9)  4.0 (Fx 2)  32.5 (Fx 13)  47.5 (Fx 19) | N/A | N/A | Mean maximum  0.745  Max proton:  0.87±0.08  1.80±0.08  0.21±0.02  0.10±0.02 | Mean Maximum 655 (330-1100)  Max neutron:  29.7  19.3  16  14.3 | Median distance in all patients: 10.0 (0.8-40.0)  Mean distance in patients with reset: 7.0 (0.9-8.0)  5.0  0.9  3.0  8.0 | ICD in 1/5 patients showed an ERI message (predicted prior to treatment - not influence by radiation)  Hardware errors: Nil  Software errors: 5  POR  POR  POR  POR  POR  All CIEDs successfully reprogrammed / No reported morbidity or mortality |

CIED = cardiac implantable electronic device; ICD = implantable cardioverter defibrillator; LIF = lead in treatment field; PM = pacemaker; PBS = pencil beam scattering; PS = passive scattering; PTV = planning target volume; CTV = clinical target volume; PER = partial electrical reset; POR = power-on reset; SOBP = Spread-out Bragg Peak; TE = transient error; ERI = elective replacement indicator; * No malfunction

**Table 1.** Continued

| **Author / Year** | **Study type / size** | **CIED type / manufacturer / year implanted** | **CIED / tumour location** | **PT mode / energy (MeV)** | **Prescribed treatment dose (GyE/Fx)** | **Cumulative dose at malfunction (GyE)** | **Dose rate (GyE min^-1^)** | **Field size /**  **beam direction** | **CIED proton dose (GyE)** | **CIED neutron dose (mSv)** | **CIED to field distance (cm)** | **Type of CIED malfunction /**  **clinical consequences** |
| --- | --- | --- | --- | --- | --- | --- | --- | --- | --- | --- | --- | --- |
| Ueyama  et al.^39^  2016 | *In vivo,* single institution  7 patients (7 PMs) | 2 PMs  *EnRhythm; Medtronic, MN, USA* **(LIF)**  *Identity Adx; St. Jude Med*  *St Jude Med  *Medtronic  *Medtronic  *Boston Scientific  *Boston Scientific | Lung  Pancreas  Larynx  Prostate  Lung  Lung  Prostate | PS / 210  PS / 150 - 210  PS / N/A  PS / N/A  PS / N/A  PS / N/A  PS / N/A | 66.0 / 10  50.0 / 25  N/A  N/A  N/A  N/A  N/A | 52.8 (Fx 8)  At simulation  26.0 (Fx 13)  N/A  N/A  N/A  N/A  N/A | N/A | N/A | N/A | 15.5  3.8  N/A  N/A  N/A  N/A  N/A | Median: 24  24  30  13  >50  17  18  >50 | Hardware errors: Nil  Software errors: 3  POR  POR  POR, emergency event  All CIEDs successfully reprogrammed / No reported morbidity or mortality |
| Seidensaal et al.^40^  2019 | *In vivo* retrospective, single institution  10 patients (~ 90% PMs, 10% ICDs) | *Biotronik (13%)  *Boston Scientific (10%)  *Medtronic (61%)  *St. Jude Med (13%)  *ELA Med (3%) | 69% H&N  19% abdo-pelvis  12% thorax  Thorax (lymphoma) | PBS / N/A | Median 51.0 (10-66) / 17.5 (5-33)  40.0 / 20 | N/A | N/A | N/A | N/A | N/A | Median distance of CIED to PTV  13.4 (4.1-17.9),  CIED to 10% isodose line 11.6 (2.4-17.1)  CIED to the proton beam (measured on beam’s eye view) 8.3cm (range 0-18cm) | 1 enhanced impedance of the pacing leads (present prior to treatment - not influence by radiation)  Note: in 2 thoracic patients (incl. the lymphoma patient) CIED to PTV distance was 4-5cm  All CIEDs successfully reprogrammed / No reported morbidity or mortality |

CIED = cardiac implantable electronic device; ICD = implantable cardioverter defibrillator; LIF = lead in treatment field; PM = pacemaker; PBS = pencil beam scattering; PS = passive scattering; PTV = planning target volume; CTV = clinical target volume; PER = partial electrical reset; POR = power-on reset; SOBP = Spread-out Bragg Peak; TE = transient error; ERI = elective replacement indicator; * No malfunction

**Table 1.** Continued

| **Author / Year** | **Study type / size** | **CIED type / manufacturer / year implanted** | **CIED / tumour location** | **PT mode / energy (MeV)** | **Prescribed treatment dose (GyE/Fx)** | **Cumulative dose at malfunction (GyE)** | **Dose rate (GyE min^-1^)** | **Field size /**  **beam direction** | **CIED proton dose (GyE)** | **CIED neutron dose (mSv)** | **CIED to field distance (cm)** | **Type of CIED malfunction /**  **clinical consequences** |
| --- | --- | --- | --- | --- | --- | --- | --- | --- | --- | --- | --- | --- |
| Bjerre et al.^41^  2021 | *In vitro*  Experimental  62 CIEDs (26 PMs, 18 ICDs, 18 CRTs)  Medtronic  PM: 5  ICD: 7  CRTs: Nil  Biotronik  PM: 10  ICD: 6  CRT-P: 3  CRT-D: 3  St. Jude Med  PM: 5  ICD: 2  CRT-P: 3  CRT-D: 5  Boston Scientific  PM: 6  ICD: 3  CRT-D: 4 | Susceptible CIEDs:  Medtronic ICDs:  *Evera S Dr*  *Visia AF MRI*  *Visia AF MRI SVR*  *Evera MRI S DR*  *Vivia XT CRT-D*  Biotronik PMs:  *Enitra 6 DR-T*  *Etrinsa 6 DR-T*  *Estella DR-T*  *Evia 6 DR-T*  Biotronik ICDs:  *Iforia 5 VR-T*  *Intica 5 DR-T*  *Intica 5 VR-T*  Biotronik CRTs:  *Enitra 8 HF-T*  *Intica 7 HF-T QP*  *Lumax 640 HF-T*  *Iforia 5 HF-T*  Boston Scientific PM:  *Proponent MRI* | Placed on top of phantom covered with isotonic saline | PBS / 161.4-214.7 | 72.0 / 36 | N/A | N/A | 10x10x10 cm^3^ / 180° posterior beam | N/A | 6.94  3.71  1.91 | Vertical distance from  distal edge of SOBP to CIEDs was 3.  Lateral distance of CIEDs to SOBP:  0.5  5.0  10.0 | Hardware errors: 5  5 Medtronic ICDs had unrecoverable battery power depletion  Software errors: 61 (majority ICDs)  60 POR in 13 Biotronik devices  Risk of device reset in Biotronik CIEDs:  6 CIEDs, 19.4%  4 CIEDs, 5.1%  4 CIEDs, 3.2%  All CIEDs except 1 Boston Scientific PM successfully reprogrammed  critical POR in 1 Boston Scientific PM locked in permanent safety mode which had be substituted |

CIED = cardiac implantable electronic device; ICD = implantable cardioverter defibrillator; LIF = lead in treatment field; PM = pacemaker; PBS = pencil beam scattering; PS = passive scattering; PTV = planning target volume; CTV = clinical target volume; PER = partial electrical reset; POR = power-on reset; SOBP = Spread-out Bragg Peak; TE = transient error; ERI = elective replacement indicator; * No malfunction

**Table 1.** Continued

| **Author / Year** | **Study type / size** | **CIED type / manufacturer / year implanted** | **CIED / tumour location** | **PT mode / energy (MeV)** | **Prescribed treatment dose (GyE/Fx)** | **Cumulative dose at malfunction (GyE)** | **Dose rate (GyE min^-1^)** | **Field size /**  **beam direction** | **CIED proton dose (GyE)** | **CIED neutron dose (mSv)** | **CIED to field distance (cm)** | **Type of CIED malfunction /**  **clinical consequences** |
| --- | --- | --- | --- | --- | --- | --- | --- | --- | --- | --- | --- | --- |
| Hashimoto et al.^42^  2021 | *In vivo*  Retrospective,  Multi-institutional  47 CIEDs (93% PMs, 6% ICDs, 1% CRT-D) | PMs:  *Affinity DR 5330; St. Jude Med*  *Integrity µ SR;*  *St Jude Med*  *Unknown*  *Philos DR; Biotronik*  ICDs:  *Lexos DR; Biotronik*  CRT-D:  *Insync III Marquis 7279; Medtronic*  *_____________*  *BostonScientific  *Guidant  *Vitatron  *Guidant  *Pacesetter  *Sorin | Liver  Liver  Lung  Mandible  Lung  Liver | PS / 155.0  PS / 210.0  PS / 200.0  PS / 150.0  PS / 155.0  PS / 155.0 | Median: in all patients 70.0 (36.3-88.0).  2.0-6.6 GyE/Fx.  66.0 / 10  67.5 / 25  72.6 / 22  70.2 / 35  70.0 / 35  72.6 / 22 | 39.6 (Fx 6)  18.9 (Fx 7)  27.0 (Fx 10)  24.0 (Fx 7)  66.0 (Fx 20)  27.0 (Fx 13)  N/A  N/A | N/A | Max field size in cm^2^  30  145  90  115  65  55 | N/A | N/A | N/A  N/A  15-20  N/A  15-20  N/A | Hardware errors: Nil  Software errors: 10  Reset (unspecified)  Reset (unspecified)  Reset (unspecified)  Reset (unspecified)  Reset (unspecified)  Reset (unspecified)  3 TE  Reset (unspecified)  All CIEDs successfully reprogrammed / No reported morbidity or mortality |

CIED = cardiac implantable electronic device; ICD = implantable cardioverter defibrillator; LIF = lead in treatment field; PM = pacemaker; PBS = pencil beam scattering; PS = passive scattering; PTV = planning target volume; CTV = clinical target volume; PER = partial electrical reset; POR = power-on reset; SOBP = Spread-out Bragg Peak; TE = transient error; ERI = elective replacement indicator; * No malfunction

**Table 1.** Continued

| **Author / Year** | **Study type / size** | **CIED type / manufacturer / year implanted** | **CIED / tumour location** | **PT mode / energy (MeV)** | **Prescribed treatment dose (GyE/Fx)** | **Cumulative dose at malfunction (GyE)** | **Dose rate (GyE min^-1^)** | **Field size /**  **beam direction** | **CIED proton dose (GyE)** | **CIED neutron dose (mSv)** | **CIED to field distance (cm)** | **Type of CIED malfunction /**  **clinical consequences** |
| --- | --- | --- | --- | --- | --- | --- | --- | --- | --- | --- | --- | --- |
| Stick et al.^43^  2022 | *In vivo*  Retrospective,  Single institution  15 patients (10 breast; 5 H&N)  5 /10 breast patients (with real CIED) treated with photon therapy. Then proton therapy plans were created for evaluation. The remaining patients (without CIED) treated with proton therapy. Virtual CIEDs delineated instead | N/A | 8 breast (contralateral virtual or real CIED)  2 breast (ipsilateral real CIED)  5 H&N (ipsilateral virtual CIED) | PBS / 139-193  PBS / 144-199 | 50.0 / 25  40.0 / 15  26.0 / 5  50.0 / 25  40.0 / 15  26.0 / 5  50-68 / 33 or 34 | N/A | N/A | CTV volume (cm^3^)  281-2977  412-955  N/A | N/A | Max. dose to CIED  1.3-8.0  1.7-10.6  3.2-20.8  Max. dose to CIED+5mm  5.6-7.2  7.4-9.6  14.5-18.6  Max. dose to CIED  2.4-4.9  Max. dose to CIED+5mm  2.7-6.3 | CIED to CTV (8.4-13.0)  CIED to 25% isodose line (3.8-9.9)  CIED to 5% isodose line (0.8-6.6)  CIED to CTV (2.0-3.1)  CIED to CTV (2.7-5.5)  CIED to 25% isodose line (0-2.4)  CIED to 5% isodose line (0-1.4) | breast patients with contralateral CIED: treatment eligibility depends on CTV size and dose per fraction  Patients with ipsilateral CIED: Not eligible if CIED to CTV distance <2cm  Patients with incoming beam through CIED: Not eligible  Not incoming beam through CIED depends on CIED to CTV distance:  <2cm not eligible  2-4cm maybe eligible  >4cm eligible |

CIED = cardiac implantable electronic device; ICD = implantable cardioverter defibrillator; LIF = lead in treatment field; PM = pacemaker; PBS = pencil beam scattering; PS = passive scattering; PTV = planning target volume; CTV = clinical target volume; PER = partial electrical reset; POR = power-on reset; SOBP = Spread-out Bragg Peak; TE = transient error; ERI = elective replacement indicator; * No malfunction
